# Perceived Utility of the RE-AIM Framework for Health Promotion/Disease Prevention Initiatives for Older Adults: A Case Study from the U.S. Evidence-Based Disease Prevention Initiative

**DOI:** 10.3389/fpubh.2014.00143

**Published:** 2015-04-27

**Authors:** Marcia G. Ory, Mary Altpeter, Basia Belza, Janet Helduser, Chen Zhang, Matthew Lee Smith

**Affiliations:** ^1^Department of Health Promotion and Community Health Sciences, School of Public Health, Texas A&M Health Science Center, College Station, TX, USA; ^2^Center for Health Promotion and Disease Prevention, University of North Carolina at Chapel Hill, Chapel Hill, NC, USA; ^3^Health Promotion Research Center, School of Nursing and School of Public Health, University of Washington, Seattle, WA, USA; ^4^Department of Health Policy and Management, School of Public Health, Texas A&M Health Science Center, College Station, TX, USA; ^5^Emory Global Health Institute, Emory University, Atlanta, GA, USA; ^6^Department of Health Promotion and Behavior, College of Public Health, The University of Georgia, Athens, GA, USA

**Keywords:** RE-AIM, program planning, program implementation, program evaluation, older adults, aging

## Abstract

Dissemination and implementation (D&I) frameworks are increasingly being promoted in public health research. However, less is known about their uptake in the field, especially for diverse sets of programs. Limited questionnaires exist to assess the ways that frameworks can be utilized in program planning and evaluation. We present a case study from the United States that describes the implementation of the RE-AIM framework by state aging services providers and public health partners and a questionnaire that can be used to assess the utility of such frameworks in practice. An online questionnaire was developed to capture community perspectives about the utility of the RE-AIM framework. Distributed to project leads in 27 funded states in an evidence-based disease prevention initiative for older adults, 40 key stakeholders responded representing a 100% state-participation rate among the 27 funded states. Findings suggest that there is perceived utility in using the RE-AIM framework when evaluating grand-scale initiatives for older adults. The RE-AIM framework was seen as useful for planning, implementation, and evaluation with relevance for evaluators, providers, community leaders, and policy makers. Yet, the uptake was not universal, and some respondents reported difficulties in use, especially adopting the framework as a whole. This questionnaire can serve as the basis to assess ways the RE-AIM framework can be utilized by practitioners in state-wide D&I efforts. Maximal benefit can be derived from examining the assessment of RE-AIM-related knowledge and confidence as part of a continual quality assurance process. We recommend such an assessment be performed before the implementation of new funding initiatives and throughout their course to assess RE-AIM uptake and to identify areas for technical assistance.

## Introduction

With concerns about the aging population and attendant growth of multiple co-morbidities (1, 2) support has grown for national initiatives to improve the health, function, and quality of life of older adults (3, 4). Despite the growing evidence base about the nature of public health problems among older adults and successful intervention approaches for improving their health and well-being (5–7), there remains a notable gap in transferring what we know works into practice (8, 9). Many reasons can be cited for the existence of a research-to-practice gap including that researchers are not aware of the realities of programmatic implementation in real world settings and community providers lack the guidance for implementing proven programs tested in other settings (10). There is also a lack of quality questionnaires for assessing programmatic implementation, especially in multi-site intervention initiatives (11).

Originally conceived in the late 1990s, the RE-AIM framework (12) was designed to assess the public health impact of health promotion interventions through the identification of five core evaluation elements (i.e., reach, efficacy/effectiveness, adoption, implementation, and maintenance). In an attempt to understand better the translation of interventions tested within controlled trials to implementation within community settings (13), RE-AIM has changed the research paradigm from one focused exclusively on controlled clinical trials with a priority on internal validity to one that acknowledges the importance of pragmatic interventions that give salience to external validity – or the degree to which intervention results can be generalized across interventions, populations, and settings (14–18). The use of the RE-AIM framework has been refined since its conception to include guidance for the planning, implementation, maintenance, and evaluation of programs and policies by clinicians, community providers, and policy makers (19). Its utilization is appropriate for those in the fields of aging services and public health, as well as allied disciplines.

Building on early community-wide efforts to identify best practice programs for older adults through the aging services network, the United States Administration on Aging (AoA), a program division within the Administration for Community Living (ACL), has dedicated resources to the implementation and dissemination of state-wide evidence-based practices (20). This emphasis on evidence-based practices reflects the emergence of several well-tested health promotion/disease prevention programs, which have been shown to not only make a difference in older adults’ health but also in reduced health care utilization (21).

In 2006, the Atlantic Philanthropies and the AoA funded the evidence-based disease prevention (EBDP) initiative with the intention of supporting stronger linkages between State Aging Services and State Health Departments to address the health needs of the growing population of older adults. The overall goals of this initiative were to (1) develop the systems necessary to support the ongoing implementation and sustainability of evidence-based programs for older adults; (2) develop multi-sector community partnerships to enhance program accessibility and extend program capacity; (3) reach the maximum number of at-risk older adults who could benefit from the programs; and (4) deliver evidence-based programs with fidelity (22).

Seen as an opportunity for fostering learning collaborative, the funders contracted for technical assistance to the 27 state grantees funded under the EBDP initiative. Since this was the first time RE-AIM was integral to health promotion program implementation activities for these partnerships, there was interest in exploring how well and in what ways the framework was being adopted and applied, especially since no systematic collection of this information existed. As investigators from three CDC Prevention Research Center–Healthy Aging Research Network (HAN) campuses charged with providing technical assistance to the funder and State grantees, we wanted to explore how translational research frameworks were being implemented in the real world settings by state-level aging services providers and their public health partners. This paper expands upon previously reported findings (23). Its purposes are to (1) introduce the reader to the RE-AIM framework; (2) describe the development of a questionnaire to assess the implementation processes in the field based on elements from the RE-AIM framework; (3) using this questionnaire, examine ways RE-AIM was viewed by grantees and used in their program planning, implementation, and evaluation of evidence-based programs; and (4) summarize implications for future use of RE-AIM and training needs in the evaluation of community-based dissemination and implementation (D&I) efforts of evidence-based programs.

## Materials and Methods

### Definitions of RE-AIM elements

**Figure 1:**
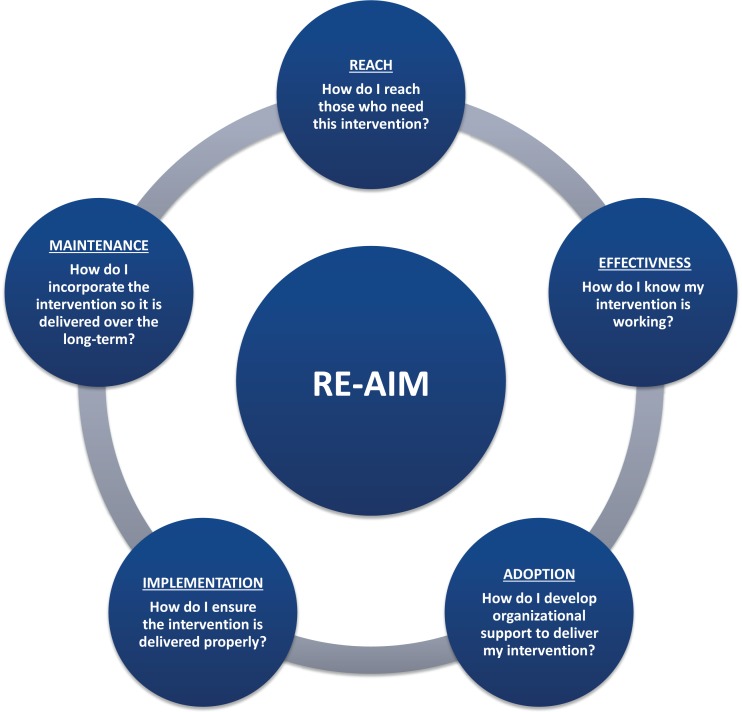
**RE-AIM elements: planning and evaluating questions (see www.re-aim.org for more information)**.

As illustrated in Figure 1, the acronym RE-AIM represents the five essential components of the RE-AIM framework: reach, effectiveness, adoption, implementation, and maintenance (24). Each component addresses a major research question that can guide program planning and evaluation.

“Reach” is the extent to which a program attracts and retains the target audience. Measures of Reach include the number, proportion, and representativeness of participants. It is important to monitor Reach to determine if the desired audience is participating in the program, in what numbers, and whether there is program completion or attrition. This in turn, can help gage the success of marketing, recruitment, and retention efforts.

“Effectiveness” refers to assessing the change in short- and/or long-term program outcomes, such as health behaviors and lifestyles, symptom management, health status, or health care utilization outcomes. Effectiveness indicators also monitor for other outcomes, whether negative or unintended that result from the program. It is important to monitor Effectiveness to provide the evidence as to whether the program is producing positive changes, which ultimately makes the case for the program’s value and return on investment.

“Adoption” activities assess organizational capacity and partnership support. Measures include the number, proportion, and representativeness of staff and settings who adopt a program as well as tracking of the various ways partners contribute to program delivery. It is important to know if the supply of delivery staff and sites matches program demand and is located in areas where the target audience resides and whether there is capacity to bring the program to scale.

“Implementation” is the extent to which the program is delivered consistently, as intended by the program developers, across all implementation sites by all instructors. Implementation measures also tracks program costs. It is important to monitor Implementation in order to identify areas of need for improvement in program delivery, assure participant results can be attributed to the program and identify return on investment for stakeholders.

At the setting level, “Maintenance” refers to the extent to which the program can be embedded within the routine organizational practice. Some factors, such as “ongoing staff support,” “partnership with community,” “sufficient funding,” and “health marketing,” are all essential elements for organizational maintenance. At the individual level, “Maintenance” refers to the extent to which individual participants experience long-term benefits (longer than 6-months following program completion) and better quality of life from the health promotion interventions or policies. Attention to these elements helps inform strategies to ensure individual benefits are sustained over time and that the necessary infrastructure is in place to ensure a program will receive ongoing institutional or community support.

### Procedures

Data were collected using internet-delivered methodology. The questionnaire utilized to collect data from respondents was developed by the HAN project team using online survey software. Electronic mail-based invitations to participate in the questionnaire were sent in January 2009 to designated project leads representing 27 states receiving funding from and participating in the EBDP initiative. The instructions requested that the questionnaire be completed separately by one state lead (either public health or aging) and one state-level program evaluator. Other team members who played key roles in program implementation and/or evaluation (e.g., a local project coordinator and/or regional coordinator or university partner) were also welcome to complete the questionnaire. Some of the items (e.g., knowledge and confidence in applying the RE-AIM framework) were asked retrospectively. After completing the questionnaire, the respondents were invited to share their responses with their state team as a way of enhancing their planning and evaluation efforts. The initial survey requested that responses be returned within 2 weeks. Two follow-up emails were sent to state respondents to increase the survey response rate. This study received Institutional Review Board (IRB) approval at Texas A&M Health Science Center where data were collected and analyzed.

### Questionnaire and measures

Reflecting expertise in several health professions (public health, nursing, and social work) and prior experience with the RE-AIM model and implementation research (25–29), the authors designed the questionnaire to address how state grantees integrated RE-AIM elements into different planning, implementation, evaluation, and monitoring processes (a copy of the full questionnaire is appended to the end of this article).

As there were no comparable questionnaires in the literature, the authors built the questionnaire around concepts deemed important to reflect implementation processes. The questionnaire was designed to collect information about the respondent’s knowledge, attitudes, and current practices related to different aspects of the RE-AIM framework as a whole as well as attention to its individual components. The questionnaire was pilot tested for ease of understanding and face validity with local community practitioners.

The final questionnaire contained 47 multi-part items including close-ended and open-ended items, as well as checklists. Recognizing the importance of “survey fatigue” or attrition, the HAN project team was careful not to make the questionnaire too long. Therefore, close-ended items with Likert-type scaling were used to make it easy for respondents to respond to questionnaire items. Additionally, open-ended items were integrated into the questionnaire to allow for additional responses to give richer detail and context to close-ended items.

It was estimated that the online survey would take approximately 10–20 minutes to complete. Individualized links were sent through the online survey website to state leads that were identified through the AoA’s Technical Assistance Center. Respondents had unlimited access to the online questionnaire to enable them to complete the task at their convenience and as a means of increasing completion rates. The questionnaire opened with a brief definition of the RE-AIM elements, with directions to the respondents to go to the RE-AIM website (www.re-aim.org) if they desired more information about the rationale for and measurement of each element.

#### RE-AIM utilization

Respondents were asked to rate the degree to which the RE-AIM framework was used for planning, implementation/evaluation, and maintenance. A series of 15 items were used to assess aspects of utilization. For example, for planning, respondents were asked to respond to how they used RE-AIM to “select community partners,” “select host and/or implementation sites,” and “select assessment/evaluation tools.” For implementation/evaluation, respondents were asked to rate the framework use for “plan or alter participant recruitment,” “conduct mid-course evaluations,” and “present/publicize program findings.” For maintenance, respondents rated the framework use for “secure funding for maintaining program delivery,” “build infrastructure to maintain program staffing,” and “build capacity for ongoing quality assurance (QA).”

#### Self-rated knowledge

Respondents were asked to rate their knowledge about “EBDP programs” and “the RE-AIM framework” at the start of the grant initiative (retrospectively) versus the current time. If respondents were not present at the initial stages of program implementation, they were instructed to mark the “not relevant” category.

#### RE-AIM-related confidence

Self-efficacy refers to individuals’ beliefs in their ability to succeed in a given situation (30). These beliefs act as determinants of how individuals think, behave, and feel (31). Individuals’ sense of self-efficacy determines how goals, tasks, and challenges are addressed. Individuals with a strong sense of self-efficacy view challenging problems as tasks to be mastered; develop stronger interest in the activities in which they participate; and are more committed to their interests. (30) We were interested in learning about grantees confidence in the use and application of RE-AIM and whether their confidence levels changed over the course of the grant. “Confidence” is the term Bandura uses as synonymous to self-efficacy when measuring the construct. Respondents were asked questions to measure their confidence about applying each of the five RE-AIM at the start of the grant initiative (retrospectively) versus the current time. Again, if respondents were not present at the initial stages of program implementation, they were instructed to mark the “not relevant” category.

#### Perceptions of RE-AIM usefulness

Respondents were asked to share their attitudes about the application of RE-AIM for various tasks related to their grant efforts. Respondents were asked to rate the usefulness of RE-AIM applied to the following activities: “planning of this initiative,” “implementation of this effort,” “evaluation of this effort,” “planning efforts with our other aging programs,” and “implementation efforts with our other aging programs.” Respondents were also asked to report how valuable they believed RE-AIM was for different audiences. Participants were asked to respond to the following audiences: “providers,” “community leaders,” “policy makers,” and “evaluators.” Finally, respondents were asked to indicate if they would apply RE-AIM in their future projects.

#### Ease of RE-AIM use and application

Respondents were asked to report how easy they believed RE-AIM was to use/apply and their preferences about monitoring RE-AIM elements. Respondents were asked to respond to seven statements about the RE-AIM framework as a whole as well as its component elements.

#### Respondent characteristics

Items were included to collect information about the respondents’ role on the AoA/Atlantic EBDP grant (i.e., state lead, state evaluator, regional project coordinator, local project coordinator, and other); the year that the respondent started working with evidence-based programs (i.e., from 2000 to 2008); and the type of evidence-based programs being delivered (from a list of 16 approved evidence-based programs).

## Results

### Utility of an online survey for collecting information in a multi-state initiative with multiple stakeholders and program types

As previously reported (23), 40 questionnaires were submitted electronically representing a 100% state-participation rate among the 27 funded grantee states. Almost half (48.2%) of the states had two respondents. Approximately one-third of the states (37.0%) reported not having a state-wide evaluator. State leads and state-wide coordinators represented the majority of respondents (65%); state-wide evaluators represented 30% of the respondents; and regional or local coordinators represented the remaining 5% of the respondents.

In terms of when they started working with EBDP programs for older adults, less than half of the respondents reported that they had worked with evidence-based programs before the onset of the current initiative. Of the 16 approved evidence-based programs, 15 programs were offered across the grantee states. The most commonly offered programs by grantee states included Chronic Disease Self-Management Program (CDSMP) (100%), EnhanceFitness (37.5%), A Matter of Balance (30.0%), and Healthy IDEAS (10.0%). There were no reported problems with understanding or answering any questionnaire items.

### Application of RE-AIM for planning, implementation/evaluation, and monitoring

Table 1 reports the extent to which respondent’s decisions about this initiative were influenced by the RE-AIM framework in terms of planning, implementation/evaluation, and maintenance. With respect to planning, the largest proportion of respondents reported RE-AIM influenced their decisions about selecting evidence-based programs to deliver, identifying target populations, and selecting assessment/evaluation tools. With respect to implementation/evaluation, about 58% of respondents reported RE-AIM influenced decisions about planning or altering participant recruitment. A majority of respondents reported RE-AIM moderately influenced decisions when conducting mid-course evaluations and structuring reports. With respect to maintenance, a majority of respondents reported RE-AIM influenced decisions about planning for program sustainability. A majority of respondents reported RE-AIM moderately influenced decisions about maintenance strategies related to participant improvement, securing funding, and ongoing QA.

**Table 1:** **Ways in which RE-AIM was used for planning, implementation/evaluation, and maintenance (*n* = 40)**.

	Not at all (%)	A little (%)	Some (%)	A lot (%)	Do not know (%)
**Planning**					
Select community partners	10.5	28.9	39.5	7.9	13.2
Select evidence-based programs for implementation	17.5	17.5	30.0	20.0	12.8
Select host and/or implementation sites	7.7	25.6	35.9	15.4	15.4
Identify target populations (people who may participate in programs)	12.5	22.5	30.0	22.5	15.0
Select assessment/evaluation tools	13.2	21.1	32.5	20.2	12.5
**Implementation Evaluation**					
Plan or alter participant recruitment	10.5	21.1	39.5	18.4	10.5
Structure agendas and/or team meetings	17.5	25.0	25.0	20.0	12.5
Conduct mid-course evaluations	10.0	25.0	30.0	22.5	12.5
Structure reports	15.0	30.0	27.5	20.0	7.5
Present/publicize program findings	12.5	22.5	22.5	27.5	15.0
**Maintenance**					
Address strategies for maintaining participant improvement	10.3	30.8	25.6	15.8	18.4
Guide discussions and/or planning around program sustainability	10.0	20.0	30.0	30.0	10.0
Secure funding for maintaining program delivery	15.8	31.6	27.5	15.0	12.5
Building infrastructure to maintain program staffing	12.5	27.5	22.5	22.5	15.0
Build capacity for ongoing quality assurance	5.1	33.3	28.2	25.6	7.7

### Knowledge and confidence with EBDP and RE-AIM elements over time

Table 2 reports respondents’ knowledge about EBDP and RE-AIM, as well as confidence applying RE-AIM elements at the start of the initiative versus the time in which they completed this study. On average from the start of the initiative to the time of the questionnaire (approximately 2 years), fewer than half of respondents increased their knowledge about EBDP programs, yet, over two-thirds increased their knowledge about the RE-AIM framework. In terms of confidence applying elements of the RE-AIM framework, the largest increase was reported for applying reach, adoption, and implementation, which was followed by maintenance and effectiveness.

**Table 2:** **Knowledge about and confidence applying RE-AIM elements at the start of the intervention versus the current time (*n* = 40)**.

	At start	Currently	Improvement (%)
Knowledge about evidence- based disease prevention programs	2.73	3.92	43.6
Knowledge about RE-AIM framework as a whole	1.98	3.33	68.2
Confidence applying the RE-AIM elements			
Reach	2.13	3.43	61.0
Effectiveness	2.13	3.13	46.9
Adoption	2.08	3.35	61.1
Implementation	2.10	3.38	61.0
Maintenance	2.05	3.26	59.0

*Items scored from, not at all (1) to a lot (4)*.

### Perceptions of RE-AIM usefulness for various tasks and audiences

Table 3 reports respondents’ attitudes about the usefulness of the RE-AIM application for various tasks and audiences. The vast majority agreed the framework was useful for planning, for implementation, and for evaluation. When asked about the application of RE-AIM in other aging programs, the majority also agreed that the framework was useful for planning and for implementation. Further, when asked about audiences for which the RE-AIM framework is most useful, the majority of respondents agreed RE-AIM was useful for evaluators, providers, community leaders, and policy makers.

**Table 3:** **Perceptions of RE-AIM usefulness for various tasks and audiences (*n* = 40)**.

	Disagree or strongly disagree (%)	Agree or strongly agree (%)	Do not know (%)
**Tasks**			
Planning in this initiative	5.0	90.0	5.0
Implementation of this initiative	2.5	90.0	7.5
Evaluation of this initiative	2.5	84.7	2.6
Planning efforts with other aging programs	5.0	85.0	10.0
Implementation efforts with other aging programs	2.5	87.5	10.0
**Audiences**			
Providers	2.5	77.5	20.0
Community leaders	2.5	77.5	20.0
Policy makers	5.0	72.5	22.5
Evaluators	0.0	92.5	7.5

### Perceptions of ease of using the RE-AIM framework and monitoring RE-AIM elements

Table 4 reports respondents’ perceptions about the ease of using and applying RE-AIM and their preferences about monitoring RE-AIM elements. Approximately three-quarters of respondents agreed that it was easy to understand the RE-AIM elements. Further, only a small minority believed that RE-AIM was too academic and took too much time to implement. However, nearly half of the respondents felt special expertise was required to monitor RE-AIM requirements and approximately one-third felt the successful application of RE-AIM elements was difficult to measure. When asked about monitoring RE-AIM elements, over half believed it was best to track all of the elements, whereas a sizable proportion of respondents (over one-third) believed looking at one or two elements was most useful.

**Table 4:** **Perceived ease to use and apply RE-AIM and preferences about monitoring RE-AIM elements (*n* = 40)**.

	Disagree or strongly disagree (%)	Agree or strongly agree (%)	Do not know (%)
The different RE-AIM elements are easy to understand	10.3	74.6	5.1
Monitoring RE-AIM elements requires special expertise	43.6	48.7	7.7
RE-AIM is too academic	75.0	10.0	15.0
RE-AIM takes too much time to implement	65.0	15.0	20.0
Measuring the successful application of different RE-AIM elements is difficult	40.0	32.5	27.5
Looking at just one or two RE-AIM elements is what I find most useful	52.5	35.0	12.5
I think it is best to try to track all of the RE-AIM elements	20.0	57.5	22.5

## Discussion

This study presents a unique real world application of how the RE-AIM framework was embedded into a national effort by aging services providers and their partners to expand the dissemination of evidence-based programing for older adults. The application of RE-AIM and other implementation and dissemination frameworks can be encouraged or mandated by funding agencies as illustrated by a prior examination of the application of RE-AIM to funding applications (32). However, little is known about how key state decision makers will actually employ different RE-AIM elements in their grant planning, implementation, and maintenance activities. Thus, this study adds to our understanding of the general use of RE-AIM for different grant tasks, and how the application and usefulness varies by specific users.

In contrast to previous research that documents a primary focus on reach and effectiveness and excludes attention to maintenance (33), in this initiative the RE-AIM framework was used by state agencies for building infrastructure or capacity for ongoing QA and sustainability. In retrospect, this is not surprising given the salience of sustainability to this initiative and targeted technical assistance from the funder and outside consultants in this area.

An important issue addressed in this research was the extent to which RE-AIM elements were seen as an indivisible whole versus the sum of individual parts. As indicated in the Section “Results,” only slightly more than half of the respondents endorsed the usefulness of tracking all of RE-AIM the elements together, while nearly a quarter did not express an opinion. It is not known if this reflects an inclination for adopting single elements over the framework as a whole, or a lack of experience with the framework, or a lack of resources to fully assess and track all of the framework components simultaneously. Additional research is needed to identify which RE-AIM components different types of program implementers will find most useful and what resources are warranted.

While there was strong endorsement of the usefulness of RE-AIM for applying various tasks, the framework was seen as most useful for evaluators versus providers, community leaders, or policy makers. This may reflect the original origins of RE-AIM as an evaluation tool for public health research (12), or the fact that about half of the respondents still felt monitoring RE-AIM elements required special expertise. Alternatively, it may be that the respondents who were evaluators in this study had more public health training. These findings point to the importance of community providers partnering with academics, with each being aware of the language and context of the other party (34). Such partnering has become even more critical with the increased push for demonstrated outcomes, continuous quality improvement (CQI) of delivery agencies, and selected funding opportunities requiring these partnerships. In community settings, it is especially important to identify and implement pragmatic measures and evaluation designs (14).

Consistent with the growth of literature about RE-AIM (19), large increases in knowledge about RE-AIM and confidence in applying the RE-AIM framework were seen over the 2-year time period from initial funding to the time of the questionnaire. It is our feeling that these large increases reflect more active dissemination versus passive diffusion of the RE-AIM framework throughout the funded states. Such increases can be attributed, in large part, to the technical assistance provided grantees about the RE-AIM framework both in terms of the annual grantee conferences as well as monthly grantee calls organized by our team. The National Council on Aging’s Center for Healthy Aging Technical Resource Center also broadly advertised and sponsored webinars and workshops featuring online self-instructional training modules that were created to train providers on how to apply the RE-AIM framework to their evidence-based health promotion programs. Many of these offerings were co-presented by academics paired with state and aging service provider partners. This enabled community respondents to receive information from peers who often served as role models in the dissemination of experience-based information about best strategies for implementing different RE-AIM elements. However, great variation in confidence improvements was observed among RE-AIM elements (i.e., 46.9% for effectiveness and 61.0% for reach, adoption, and implementation). This finding suggests that the need for additional attention for effectiveness and outcome evaluation during trainings and in online resources provided to grantees in future initiatives. Thus, we offer the questionnaire as a practical tool for collecting information about program implementation and evaluation processes from key program decision makers in a national EBDP initiative. A copy of the questionnaire is located at the end of this article.

A few limitations can be noted. With only 40 responses, this research is best viewed as an implementation case study of the RE-AIM framework. While we had anticipated having two respondents per state to reflect both planning and evaluation perspectives, it became evident that not all states had state-wide evaluators. With the small number of respondents, we were not able to examine responses by respondent type, which in turn made us unable to assess differences in perceptions by whether the respondent was a state lead, a state-wide coordinator, or program evaluator. However, it should be noted that there was representation from each of the funded states and this type of data related to practitioner self-reported confidence levels about RE-AIM use is rarely evaluated and/or reported. With the intent of collecting data from stakeholders in 27 states quickly and inexpensively, we were restricted to survey methodology. Our questionnaire reveals interesting observations about the utility of employing the RE-AIM framework, which points to issues that can be followed-up about through more in-depth interviews in a particular state.

Additionally, another potential limitation is that this current study examines a community grants program implemented at one point in time. Requests for respondents to reflect back on their familiarity and knowledge about evidence-based programs and the RE-AIM framework may be subject to recall bias or be affected by personnel changes. Hence, we recommend that implementation assessments be ongoing from the beginning to the end of the program period. Further, different intervention programs could have been implemented over time, thus, knowledge, attitudes, and practices about RE-AIM elements may be changing. Since this initial AoA EBDP initiative there has been a 2010–2012 ARRA initiative for further disseminating the CDSMP in 45 states, the District of Columbia, and Puerto Rico. However, no systematic data on the application of RE-AIM elements were collected, and the current study is the only national examination of the implementation and adoption of the RE-AIM framework in the aging services network.

### Implications for practice

We offer our questionnaire as a pragmatic tool that can be used to assess implementation of the RE-AIM framework as a whole, or its constituent parts. We recommend attention to the full continuum of implementation processes from planning, implementation, evaluation, and sustainability considerations. Additionally, users of this questionnaire will need to consider in advance the most feasible administration (e.g., by online questionnaire or in-person or telephonic interview) and ideal assessment points (e.g., before a program starts, at a midway point, and then toward the end of the program). For those interested in more comprehensive evaluation aspects, questions can also be added to determine what types of standardized outcome measures would be feasible to collect in the dissemination of EBDPs conducted outside of a research setting. Seeking such input from the field aligns with the recent emphasis on person-centered research, which stresses the importance of including major stakeholders in research (35).

As the EBDP field has matured, there are several important implications for the future use of RE-AIM. The AoA’s guidelines for initiatives in evidence-based programing for older adults now embed RE-AIM within a larger CQI approach for QA. To carry out CQI, state agencies and their partners need to orient the team about the QA plan; agree upon RE-AIM performance indicators; specify designated roles, responsibilities, and timelines for all program partners; establish mechanisms for periodic review and standardize protocols for making corrective actions when necessary (36). We believe the questionnaire we developed is valuable for conducting initial assessments, as well as ongoing assessments of the implementation and evaluation process as it unfolds over the life of a funded project.

In 2012, the U.S. ACL/AoA funded 22 states to continue to scale the evidence-based CDSMP and establish a sustainable infrastructure for EBDP program delivery (37). With QA as a central focus of the infrastructure operations, the RE-AIM framework provides the guidance for state agencies to create a comprehensive system for describing, measuring, and evaluating program delivery to ensure that respondents receive effective, quality services and that funding requirements are met. However, with the growing expansion of community partnerships for program delivery and staff turnover, ongoing training on the use of the RE-AIM framework is needed.

To support these efforts, the NCOA Center for Healthy Aging (38), building on general materials provided by the original RE-AIM developers (24), offers a myriad of tools, checklists, issue briefs, and 10 online training modules to inform and guide providers working with older populations on the application of the RE-AIM framework. Trainings about frameworks like RE-AIM would be best attended by community partners along with their academic partners to help integrate evaluation strategies and measures within the fabric of program delivery. Additional questionnaires are available now to help in the identification and selection of appropriate frameworks to inform one’s work (39), and these questionnaires could be incorporated into trainings.

Within a relatively short period of time, evidence-based health promotion programing for older adults has evolved into a system change movement with the goal of embedding these programs into integrated community, long-term care, and health systems. According to the AoA (37), state aging services and their public health partners are developing sustainable service systems utilizing diverse strategies including embedding programs within Affordable Care Act initiatives such as care transitions and medical homes; partnering with Medicaid and other health insurance providers; pursuing accreditation and Medicare reimbursement for Diabetes Self-Management Training; collaborating with Federally Qualified Health Centers, Veterans Administration Medical Centers, and other healthcare organizations; and teaming up with non-traditional partners such as the State Department of Corrections and State and Local mental health agencies. The breadth and diversity of these efforts and partnerships calls for continued attention to capacity-building through ongoing development of state-of-the-art training to address the new ways of offering evidence-based programs within an implementation and dissemination framework.

## Conflict of Interest Statement

The authors declare that the research was conducted in the absence of any commercial or financial relationships that could be construed as a potential conflict of interest.

This paper is included in the Research Topic, “Evidence-Based Programming for Older Adults.” This Research Topic received partial funding from multiple government and private organizations/agencies; however, the views, findings, and conclusions in these articles are those of the authors and do not necessarily represent the official position of these organizations/agencies. All papers published in the Research Topic received peer review from members of the Frontiers in Public Health (Public Health Education and Promotion section) panel of Review Editors. Because this Research Topic represents work closely associated with a nationwide evidence-based movement in the US, many of the authors and/or Review Editors may have worked together previously in some fashion. Review Editors were purposively selected based on their expertise with evaluation and/or evidence-based programming for older adults. Review Editors were independent of named authors on any given article published in this volume.

## Appendix

### Survey of RE-AIM Awareness and Utilization

This questionnaire is being sent to all states receiving AoA or The Atlantic Philanthropies (Atlantic) funding as part of the Evidence-Based Disease Prevention Program. We request one state lead (either public health or aging) and one program evaluator, preferably someone who works at a state-wide level to complete the questionnaire separately. Other team members who play key roles in program implementation and/or evaluation (e.g., a local project coordinator and/or regional coordinator or University partner) are also welcome to complete the questionnaire.

The purposes of this questionnaire are to (1) describe grantees awareness about RE-AIM; (2) examine ways RE-AIM is used by grantees in their program planning, implementation, and evaluation of evidence-based programs; and (3) identify useful RE-AIM materials and training needs. Information learned from this questionnaire will help the AoA and NCOA provide better assistance to grantees. Completing the questionnaire also allows state teams to reflect on issues related to program planning and implementation and use these insights to improve state and local processes.

RE-AIM is a framework that has been used in the aging services field to bridge the gap between research and practice by identifying key steps involved in the application of programs and policies in real-world settings. The five elements of RE-AIM are reach, effectiveness, adoption, implementation, and maintenance.

We recognize that each of the respondents may not be familiar with the technical items about RE-AIM or details about program implementation or assessment. If you do not know the answer to a specific question, please check the “do not know category.” Other questions are asking for attitudes about RE-AIM, and we welcome everyone’s opinion.

The questionnaire takes about 10–20 min to complete. Please complete the questionnaire by XXX. A Word document is available should you want to see all the questions in advance. While we are asking that each state respondent fill out the questionnaire independently, we suggest that the state teams may want to review their responses at a team meeting after submission.

Contact XXX for questions about the questionnaire or to obtain a Word document of the questionnaire. The questionnaire is set up such that your role and responses determine specific questions you are asked to complete (so note that the computer version may differ slightly from the word version).

Completing this questionnaire is voluntary. The responses will be confidential and reporting will occur in aggregate for the entire group of respondents. For those willing to participate further, we will also be seeking to record some in-depth experiences and will be documenting a few grantee stories that detail the successes and challenges in the application of RE-AIM elements. Thank you for your time and interest.

**I have read and understand the information above and wish to voluntarily participate in this survey**.

❑ *Yes*
  ❑ *No*

**Information about the Person Completing this Survey**

**First Name:**
_______________________________________
**Last Name:**
_______________________________________
**What is your email address:**
_______________________________________
**What is your primary role on the AoA/Atlantic evidence-based disease prevention grant project? (Select one)**
  ❑ *State lead or state-wide coordinator*
  ❑ *State-wide evaluator*
  ❑ *Regional project coordinator*
  ❑ *Local project coordinator*
  ❑ *Other*
**If you selected Other, please specify:**
___________________________________________________________
**If you selected State lead or State-wide coordinator, please specify the name of your agency:**
___________________________________________________________
**If you selected State-wide evaluator, please specify the name of your agency:**
___________________________________________________________
**May we contact you if any responses are unclear or elaboration is needed?**
  ❑ *Yes*
  ❑ *No*
**Which state are you completing this survey for?**
  ❑ *Arizona*
  ❑ *Arkansas*
  ❑ *California*
  ❑ *Colorado*
  ❑ *Connecticut*
  ❑ *Florida*
  ❑ *Hawaii*
  ❑ *Idaho*
  ❑ *Illinois*
  ❑ *Indiana*
  ❑ *Iowa*
  ❑ *Maine*
  ❑ *Maryland*
  ❑ *Massachusetts*
  ❑ *Michigan*
  ❑ *Minnesota*
  ❑ *New Jersey*
  ❑ *New York*
  ❑ *North Carolina*
  ❑ *Ohio*
  ❑ *Oklahoma*
  ❑ *Oregon*
  ❑ *Rhode Island*
  ❑ *South Carolina*
  ❑ *Texas*
  ❑ *Washington*
  ❑ *West Virginia*
  ❑ *Wisconsin*
  ❑ *Other, please specify*
**Which evidence-based programs are you currently delivering under the auspices of the AoA/Atlantic Evidence-based Disease Prevention Program (Check all that apply)**
  ❑ *Chronic Disease Self-Management Program (CDSMP)*
  ❑ *A Matter of Balance/Volunteer Lay Leader*
  ❑ *Active Choices*
  ❑ *Active Living Every Day (ALED)*
  ❑ *Enhance Fitness*
  ❑ *Enhance Wellness*
  ❑ *Healthy Eating*
  ❑ *Healthy IDEAS*
  ❑ *Healthy Moves*
  ❑ *Medication Management*
  ❑ *PEARLS*
  ❑ *Spanish Arthritis Self-Management Program*
  ❑ *Step by Step*
  ❑ *Stepping On*
  ❑ *Strong for Life*
  ❑ *Tai Chi*
  ❑ *Other*
  ❑ *Do not know*
**If Other, please specify:**
**When did you start working with evidence-based disease prevention programs for older adults?**
  ❑ *Before 2000*
  ❑ *2001*
  ❑ *2002*
  ❑ *2003*
  ❑ *2004*
  ❑ *2005*
  ❑ *2006*
  ❑ *2007*
  ❑ *2008*

**Familiarity of and Confidence with RE-AIM**. These next set of questions pertain to your AoA/Atlantic evidence-based disease prevention state-grant funded in 2006, 2007, 2008):

**At the start of your state-grant funding (2006, 2007, 2008): Use not relevant (NR), if YOU WERE NOT present during the initial stages of the program implementation**
**Table:**
  	*not at all*	*a little*	*some*	*a lot*	*NR*
  How familiar were you with evidence-based disease prevention programs?	❑	❑	❑	❑	❑
  How knowledgeable were you with the RE-AIM framework as a whole?	❑	❑	❑	❑	❑
  How confident were you at the start of your grant-funding in applying the RE-AIM element:					
  **Reach**?	❑	❑	❑	❑	❑
  **Effectiveness**?	❑	❑	❑	❑	❑
  **Adoption**?	❑	❑	❑	❑	❑
  **Implementation**?	❑	❑	❑	❑	❑
  **Maintenance**?	❑	❑	❑	❑	❑
**At the current time**
**Table:**
  	*not at all*	*a little*	*some*	*a lot*	*NR*
  How knowledgeable are you now about evidence-based disease prevention programs?	❑	❑	❑	❑	❑
  How knowledgeable are you about RE-AIM framework as a whole?	❑	❑	❑	❑	❑
  How confident are you that you can now apply the RE-AIM element:					
  **Reach?**	❑	❑	❑	❑	❑
  **Effectiveness**?	❑	❑	❑	❑	❑
  **Adoption**?	❑	❑	❑	❑	❑
  **Implementation**?	❑	❑	❑	❑	❑
  **Maintenance**?	❑	❑	❑	❑	❑

**Application of RE-AIM**

**We are interested in learning about ways that RE-AIM has been used by the State teams in this AoA/Atlantic initiative. To what extent have decisions about the following been influenced by the RE-AIM framework and approach? Please mark DK for Do not Know**.

**Table:**
  	*not at all*	*a little*	*some*	*a lot*	*DK*
  **PLANNING:**					
  Select community partners	❑	❑	❑	❑	❑
  Select evidence-based programs for implementation	❑	❑	❑	❑	❑
  Select host and/or implementation sites	❑	❑	❑	❑	❑
  Identify target populations (people who may participate in your program[s])	❑	❑	❑	❑	❑
  Select assessment/evaluation tools	❑	❑	❑	❑	❑
  **IMPLEMENTATION/EVALUATION:**					
  Plan or alter participant recruitment	❑	❑	❑	❑	❑
  Structure agendas and/or team meetings	❑	❑	❑	❑	❑
  Conduct midcourse evaluations	❑	❑	❑	❑	❑
  Structure reports	❑	❑	❑	❑	❑
  Present/publicize program findings	❑	❑	❑	❑	❑
  **MAINTENANCE:**					
  Address strategies for maintaining participant improvement	❑	❑	❑	❑	❑
  Guide discussions and/or planning around program sustainability	❑	❑	❑	❑	❑
  Secure funding for maintaining program delivery	❑	❑	❑	❑	❑
  Building infrastructure to maintain program staffing	❑	❑	❑	❑	❑
  Build capacity for ongoing quality assurance	❑	❑	❑	❑	❑
**What other decisions have been influenced by the RE-AIM framework and approach?**
**——————————————————————————————————**

**Attitudes regarding Application of RE-AIM. Please base your responses in terms of your attitudes related to the application of RE-AIM in your AoA/Atlantic evidence-based disease prevention project**

**What is your level of agreement with each of the following:**
**RE-AIM is useful for:**
**Table:**
  	*strongly*	*Disagree*	*agree*	*strongly*	*DK*
  	*disagree*			*agree*	
  Planning in this initiative	❑	❑	❑	❑	❑
  Implementation of this effort	❑	❑	❑	❑	❑
  Evaluation of this effort	❑	❑	❑	❑	❑
  Planning efforts with our other aging programs	❑	❑	❑	❑	❑
  Implementation efforts with our other aging programs	❑	❑	❑	❑	❑
**How valuable do you see RE-AIM for different audiences? What is your level of agreement with each of the following?**
**RE-AIM is a valuable tool for:**
**Table:**
  	*strongly*	*disagree*	*agree*	*strongly*	*DK*
  	*disagree*			*agree*	
  Providers	❑	❑	❑	❑	❑
  Community Leaders	❑	❑	❑	❑	❑
  Policy makers	❑	❑	❑	❑	❑
  Evaluators	❑	❑	❑	❑	❑
**What is your level of agreement with each of the following statements about RE-AIM?**
**Table:**
  	*strongly*	*disagree*	*agree*	*strongly*	*DK*
  	*disagree*			*agree*	
  RE-AIM is too academic	❑	❑	❑	❑	❑
  Monitoring RE-AIM elements requires special expertise	❑	❑	❑	❑	❑
  The different RE-AIM elements are easy to understand	❑	❑	❑	❑	❑
  The training I have received in how to apply RE-AIM is sufficient	❑	❑	❑	❑	❑
  RE-AIM takes too much time to implement	❑	❑	❑	❑	❑
  Measuring the successful application of different RE-AIM elements is difficult	❑	❑	❑	❑	❑
  Looking at just one or two RE-AIM elements is what I find most useful	❑	❑	❑	❑	❑
  I think it is best to try to track all of the RE-AIM elements	❑	❑	❑	❑	❑
  The training material explaining RE-AIM are easy to access	❑	❑	❑	❑	❑
**Does your state team measure RE-AIM elements?**
  ❑ *Yes*
  ❑ *No*
  ❑ *Do not know*
**Please indicate how *Reach* is being measured? (Check all that apply)**
  ❑ *Number of enrollees*
  ❑ *Participant characteristics*
  ❑ *Other*
**If you selected Other, please specify:**
**Please indicate how *Effectiveness* is being measured? (Check all that apply)**
  ❑ *Health status*
  ❑ *Quality of life*
  ❑ *Symptomatology (e.g., pain or fatigue)*
  ❑ *Health behaviors (physical activity or nutrition)*
  ❑ *Self-efficacy*
  ❑ *Health care utilization*
  ❑ *Health care costs*
  ❑ *Interference with routine activities*
  ❑ *Medication management*
  ❑ *Communication with health care providers*
  ❑ *Physical functioning*
  ❑ *Other*
**If you selected Other, please specify:**
**Please indicate how *Adoption* is being measured? (Check all that apply)**
  ❑ *Number of implementation sites*
  ❑ *Type of sites*
  ❑ *Location of sites*
  ❑ *Other*
**If you selected Other, please specify:**
**Please indicate how *Implementation* is being measured? (Check all that apply)**
  ❑ *Checklists*
  ❑ *Observational data*
  ❑ *Regular phone calls for retraining*
  ❑ *Periodic face to face meetings for retraining*
  ❑ *Other*
**If you selected Other, please specify:**
**Please indicate how *Maintenance* is being measured? (Check all that apply)**
  ❑ *On-going benefits for participants*
  ❑ *Continuation of program delivery*
  ❑ *Expansion of organizational partners*
  ❑ *Identification of external funding*
  ❑ *Identification of in-kind resources*
  ❑ *Other*
**If you selected Other, please specify:**

**Training Feedback and Needs**

**There are a number of resources available to help learn about RE-AIM. How valuable have these resources been to you?**
**Table:**
  	*Do not know*	*Not at all*	*A little*	*Some*	*A lot*
  	*resource*				
  **Publications and Webinars**					
  **R**e-aim.org website	❑	❑	❑	❑	❑
  *Moving Ahead: Strategies and Tools to Plan, Conduct, and Maintain Effective Community-Based Physical Activity Programs for Older Adults* (blue cover monograph, produced by HAN)	❑	❑	❑	❑	❑
  *RE-AIM for Program Planning: Overview and Applications* (NCOA issue brief)	❑	❑	❑	❑	❑
  NCOA evidence-based online training modules	❑	❑	❑	❑	❑
  NCOA issue briefs on EBHP	❑	❑	❑	❑	❑
  **Presentations and Centers**					
  Presentations about RE-AIM at national meetings	❑	❑	❑	❑	❑
  Presentations about RE-AIM at state meetings	❑	❑	❑	❑	❑
  NCOA Center for Healthy Aging Technical Resource Center	❑	❑	❑	❑	❑
**What other resources have you used to help learn about RE-AIM?**
**Is there RE-AIM training and/or technical assistance available to all of the designated geographic areas in your state grant?**
  ❑ *Yes*
  ❑ *No*
  ❑ *Do not know*
**Is this a one-time offering?**
  ❑ *Yes*
  ❑ *No*
  ❑ *Do not know*
**About how many times do you offer this training a year?**
  ❑ *2 times*
  ❑ *3-5 times*
  ❑ *6-10 times*
  ❑ *more than 10*
**Has training been helpful?**
  ❑ *Yes*
  ❑ *No*
  ❑ *Do not know*
**In what ways could the training be improved? (Check all that apply)**
  ❑ *Training on the RE-AIM components*
  ❑ *Training on the application of RE-AIM components*
  ❑ *More in-depth training on RE-AIM components*
  ❑ *Announce training in advance*
  ❑ *Offer repeated trainings*
  ❑ *Make training more practical and less academic*
  ❑ *Provide case examples from the field*
  ❑ *Conduct phone webinars*
  ❑ *Develop on-line training*
  ❑ *Other*
**If you selected Other, please specify:**
**In your state, who is currently coordinating training and technical assistance on RE-AIM? (Check all that apply)**
  ❑ *State lead(s)*
  ❑ *Program evaluator*
  ❑ *Regional coordinator*
  ❑ *Local coordinator*
  ❑ *Do not know*
  ❑ *No one*
  ❑ *Other*
**If Other, please specify:**
**In your state, who would you recommend to coordinate training and technical assistance on RE-AIM? (Check all that apply)**
  ❑ *State lead(s)*
  ❑ *Program evaluator*
  ❑ *Regional coordinator*
  ❑ *Local coordinator*
  ❑ *Do not know*
  ❑ *No one*
  ❑ *Other*
**If Other, please specify:**

**Dissemination of RE-AIM**

**Have you used RE-AIM in programmatic efforts other than the AoA/Atlantic evidence-based disease prevention programs?**
  ❑ *Yes*
  ❑ *No*
**If yes, how many different projects? (Please indicate a number)**
_______________________
**Have you taught someone else in your agency how to use RE-AIM?**
  ❑ *Yes*
  ❑ *No*
**Would you apply RE-AIM in future projects?**
  ❑ *Definitely yes*
  ❑ *Probably yes*
  ❑ *Probably no*
  ❑ *Definitely no*

**We are interested in knowing and documenting how long it is taking sites to implement programmatic activities and develop data collection systems. Please estimate the month/date that your site initiated the different activities listed below in terms of CDSMP**

**Since receiving your State funding for AoA/Atlantic evidence-based disease prevention funding (2006, 2007, or 2008)**.
**Please enter in the form of XX/XXXX (e.g., 06/2006) or enter in NR if not yet conducted. If you are not exactly sure, please give us your best estimate**.
**Table:**
  What month/year did your State offer the first CDSMP training for master trainers to work on the State evidence-based grant?	_______________________
  What month/year did you have your first lay leader training?	_______________________
  What month/year did you offer your first CDSMP class?	_______________________
  What month/year did you start collecting outcome or data?	_______________________
  What month/year did you begin analyzing your data?	_______________________
  What month/year did you provide your first report back to your community settings?	_______________________

**Current Program and Evaluation Stage Outcome Assessments**

*An outcome assessment measures programmatic impacts on each participant, e.g., on health or health behaviors, functioning or quality of life. We are interested in learning about your outcome assessments in your AoA/Atlantic Program*.

**Do you collect participant outcomes data?**
  ❑ *Yes*
  ❑ *No*
**On average, how long does it take a participant to complete current baseline outcome measures?**
  ❑ *Less than 5 minutes*
  ❑ *6-10 minutes*
  ❑ *11-20 minutes*
  ❑ *21-30 minutes*
  ❑ *More than 30 minutes*
  ❑ *Do not know*
**On average, how long does it take to complete each follow up measure?**
  ❑ *Less than 5 minutes*
  ❑ *6-10 minutes*
  ❑ *11-20 minutes*
  ❑ *21-30 minutes*
  ❑ *More than 30 minutes*
  ❑ *Do not know*
**Have you made modifications in your participant outcome assessment form in year two or three of your funding? (Check all that apply)**
  ❑ *Kept the same items, no modifications*
  ❑ *Added new items*
  ❑ *Eliminated many of the items*
  ❑ *Changed the original items*
  ❑ *Do not know*
**What were the reasons for making these changes? (Check all that apply)**
  ❑ *Questions were confusing*
  ❑ *Survey took too long*
  ❑ *We wanted to compare our findings with other states*
  ❑ *Data we were collecting was not useful*
  ❑ *Too burdensome for participants*
  ❑ *Too burdensome for staff*
  ❑ *We wanted to have data to report to our key stakeholders*
  ❑ *Wanted to wait until got programs up and running*
  ❑ *Added the common core battery recommended by Measures of Success group*
  ❑ *Other. Please Specify*

**Post Grant Data Collection Plans**

***Process data** includes demographics characteristics, number of participants, record of their attendance, characteristics of implementation sites, etc*.

***Outcome data** is programmatic effects on health, health behaviors, functioning and quality of life, etc*.

**After your AoA/Atlantic project funding ends, will you:**
**This questions pertains to *Process Data***
  ❑ *Collect approximately the same amount of process data*
  ❑ *Decrease amount of process data collected*
  ❑ *Increase amount of process data collected*
  ❑ *Not collect any process data*
  ❑ *No decision has been made yet*
  ❑ *Do not know*
**After your AoA/Atlantic project funding ends, will you:**
**This questions pertains to *Outcome Data***
  ❑ *Collect approximately the same amount of outcome data*
  ❑ *Decrease amount of outcome data collected*
  ❑ *Increase amount of outcome data collected*
  ❑ *We do not collect any outcome data now*
  ❑ *No decision has been made yet*
  ❑ *Do not know*
**In future studies what is the longest/maximum amount of time you would recommend for the collection of participant outcome data?**
  ❑ *Less than 5 minutes*
  ❑ *6-10 minutes*
  ❑ *11-20 minutes*
  ❑ *21-30 minutes*
  ❑ *More than 30 minutes*
  ❑ *Do not know*
**What is the single most important lesson learned so far about RE-AIM, the one thing you wish you would have known ahead of time?**

**You have now completed the survey. Kudos to you! Thank you for your time and interest. Click submit**.

**If you have a story you would like to share with us about your successes and/or challenges with RE-AIM, please indicate here your willingness for us to contact you**
  ❑ *You may contact me*
